# Efavirenz exposure, alone and in combination with known drugs of abuse, engenders addictive-like bio-behavioural changes in rats

**DOI:** 10.1038/s41598-018-29978-3

**Published:** 2018-08-27

**Authors:** Marisa Möller, Jaco Fourie, Brian H. Harvey

**Affiliations:** Center of Excellence for Pharmaceutical Sciences, School of Pharmacy, North West University, Potchefstroom, South Africa

## Abstract

Efavirenz is abused in a cannabis-containing mixture known as Nyaope. The addictive-like effects of efavirenz (5, 10 and 20 mg/kg) was explored using conditioned place preference (CPP) in rats following sub-acute exposure vs. methamphetamine (MA; 1 mg/kg) and Δ^9^-tetrahydrocannabinol (THC; 0.75 mg/kg). The most addictive dose of efavirenz was then compared to THC alone and THC plus efavirenz following sub-chronic exposure using multiple behavioural measures, viz. CPP, sucrose preference test (SPT) and locomotor activity. Peripheral superoxide dismutase (SOD), regional brain lipid peroxidation and monoamines were also determined. Sub-acute efavirenz (5 mg/kg) had a significant rewarding effect in the CPP comparable to MA and THC. Sub-chronic efavirenz (5 mg/kg) and THC + efavirenz were equally rewarding using CPP, with increased cortico-striatal dopamine (DA), and increased lipid peroxidation and SOD. Sub-chronic THC did not produce CPP but significantly increased SOD and decreased hippocampal DA. Sub-chronic THC + efavirenz was hedonic in the SPT and superior to THC alone regarding cortico-striatal lipid peroxidation and sucrose preference. THC + efavirenz increased cortico-striatal DA and decreased serotonin (5-HT). Concluding, efavirenz has dose-dependent rewarding effects, increases oxidative stress and alters regional brain monoamines. Efavirenz is hedonic when combined with THC, highlighting its abuse potential when combined with THC.

## Introduction

More than 56% of people living with human immunodeficiency virus (HIV) receive antiretroviral therapy (ART), which consists primarily of two nucleoside reverse transcriptase inhibitors (NRTI’s) such as zidovudine and lamivudine, and one non-nucleoside reverse transcriptase inhibitor (NNRTI) of which efavirenz ((4 S)-6-chloro-4-(2-cyclopropylethynyl)-4-(trifluoromethyl)-2,4-dihydro-1H-3,1-benzoxazin-2-one) is the most popular^1^. Efavirenz is associated with a range of neuropsychiatric side effects (see^2^ for review), with manic episodes, euphoria and dissociative effects particularly noteworthy^1,3,4^. Efavirenz is a lipophilic compound^5^, which may underlie its penchant for mediating a diverse range of CNS manifestations^2^. Preclinical studies indicate that efavirenz acts as a weak partial agonist at the serotonin 5-HT_2A_ receptor^6^, while also presenting with actions at dopamine (DA) and 5-HT transporters, thus in line with other drugs of abuse^7,8^.

Crushing and smoking of an efavirenz- cannabis mixture, commonly known as “Nyaope” or “Woonga”^9^, in certain countries like South Africa, has seen the recreational use and abuse of efavirenz escalate to alarming proportions^9^. With other HIV drugs, like dolutegravir^10^, also noted for presenting with a similar neuropsychiatric side-effect profile, there is an urgent need to identify possible mechanisms that might relate to the rewarding effects and addictive-like/abuse potential of these drugs and as such to devise possible treatments.

Addictive disorders share a number of common neurobiological substrates critical in reward and reinforcement, in particular the mesocorticolimbic DA pathway^11^, as well as 5-HT and noradrenalin (NA) related processes^12^. In fact, all major classes of drugs of abuse increase the levels of these monoamines in the frontal cortex, striatum and hippocampus, whereby they are purported to mediate euphoria, arousal, reward and relapse in humans^13,14^. However, drugs such as cocaine and methamphetamine (MA) are pro-oxidants, thereby implicating disordered redox pathways not only in the development of addiction^15^ but also mediating neuronal damage so often engendered by the abuse of these drugs^11^.

Exposure of rats to efavirenz (10 and 30 mg/kg) has been found to significantly decrease exploratory behaviour, although without evidence for addictive-like effects^6^, while another study described anxiogenic and depressogenic effects following acute and sub-chronic doses of efavirenz (25 and 50 mg/kg, respectively)^16^. The authors also found that acute doses of efavirenz (25 mg and 50 mg/kg) increased striatal DA, 5-HT and NA, with sub-chronic exposure having the opposite effect^16^. Thus, dose related neurochemical and psychotropic effects are evident with efavirenz, at least in animals. However, it’s addictive like properties with relation to dose and in comparison to known drugs of abuse, and its ability to bolster the actions of the latter drugs, requires further study.

The aim of this study was therefore to assess dose-dependent rewarding and addictive-like properties of efavirenz in a sub-acute study compared to MA and delta-9-tetrahydrocannabinol (THC), utilizing the conditioned place preference (CPP) test. Using the most effective dose, efavirenz was then studied in a sub-chronic exposure paradigm versus THC. The latter was chosen due to the often abused combination of efavirenz and cannabis^9^. Alterations in addictive, hedonic and locomotor behaviour were assessed using the CPP test, sucrose preference test (SPT) and open field test (OFT) respectively, together with assay of frontal cortical, striatal and hippocampal monoamines. Additionally, changes in brain lipid peroxidation and peripheral redox status by assessing superoxide dismutase (SOD) activity. Lastly, combined sub-chronic efavirenz plus THC exposure was investigated to establish any possible augmenting actions with regard to the above responses.

## Material and Methods

### Statement on ethics

The study and article were conducted and presented according to the Animal Research: Reporting of *In Vivo* Experiments (ARRIVE) guidelines, as previously described^17^. The animals were handled according to the code of ethics in research, training and testing of drugs in South Africa with appropriate ethical approval (NWU - 00267- 16- S5; North-West University animal research ethics committee (AnimCare); NHREC reg. number AREC-130913-015).

### Animals

Male adult Sprague-Dawley (SD) rats, weighing 200g – 250g at the beginning of drug exposure (Vivarium, North West University), were randomized into 12 rats per exposure group with a total of 12 groups (n = 84 for the sub-acute study and n = 60 for the sub-chronic study). The rats were housed under identical conditions in the vivarium (SAVC reg. no. FR15/13458; SANAS GLP compliance no. G0019): cages (230(h) × 380(w) × 380(1) mm)) with corncob, temperature (21 ± 2 °C), humidity (50 ± 10%), white light (350–400 lux), 12 h light/dark cycle and free access to food and water^18^.

### Study design

**Figure 1:**
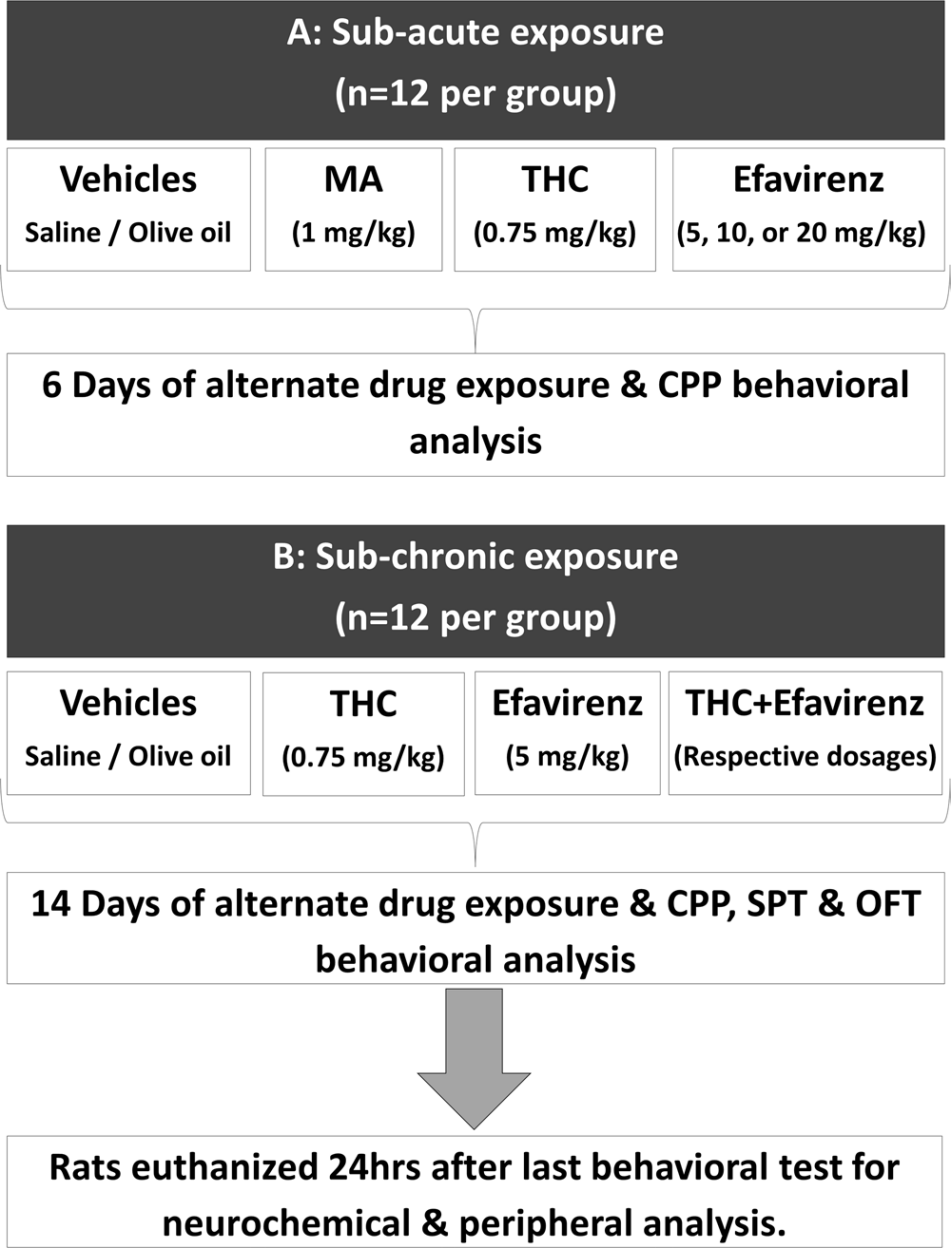
Study design: (**A**) Sub-acute study with 7 groups of rats (n = 12 rats per group) exposed to indicated drugs at alternate day dosing for 6 days and the conditioned place preference (CPP) test performed after 6 days of conditioning. (**B**) Sub-chronic study with 5 groups of rats (n = 12 rats per group) exposed to indicated drugs at alternate day dosing for 14 days during which the CPP test, open field test (OFT) and the sucrose preference test (SPT) were performed. Regional neurochemical analysis was performed in the frontal cortex, striatum and hippocampus and peripheral analysis in the plasma.

This study comprises a sub-acute and sub-chronic cohort. The former is a dose-finding behavioural study for later application to evaluate the possible enduring changes in the behavioural and neurobiological response of long-term exposure to efavirenz alone and in combination with THC. The sub-acute study consisted of 7 exposure groups receiving either MA (1 mg/kg)^19^, THC (0.75 mg/kg)^20^, efavirenz (at 5, 10 and 20 mg/kg), vehicle for MA (saline) and vehicle for THC and efavirenz (pharmaceutical grade olive oil), as illustrated in Fig. 1A. The sub-chronic study consisted of 5 groups receiving either efavirenz (at the most rewarding dosage as determined in the sub-acute study), THC (0.75 mg/kg), THC + efavirenz or vehicle (saline and olive oil respectively), as indicated in Fig. 1B. A saline control was also introduced in the sub-chronic study in order to rule out any potential antioxidant effects of olive oil as a vehicle for THC and efavirenz. All animals were randomly assigned to a specific drug exposure group, making use of a block randomization method^21^. Drug exposure lasted for 6 days in the sub-acute study and 14 days in the sub-chronic study (with alternate day dosing) for the CPP paradigm^6,20^. The sub-chronic study also included the following behavioural analyses: the OFT (day 13 of exposure) and the SPT (day 4 and 14 of exposure). All animals were euthanized (via decapitation) 24 hrs after the last behavioural test (CPP post-conditioning test) in the sub-chronic study, whereupon regional brain tissue as well as trunk blood were collected for later assay.

### Drugs and drug exposure protocol

All drugs and vehicles were injected intra-peritoneally (i.p) at 9:00 am^6,19,20^. Vehicle, consisting of saline and pharmaceutical grade olive oil and adjusted to an average physiological pH, was administered on the days of vehicle exposure. Olive oil was used as a vehicle for both THC and efavirenz, which are lipophilic drugs^22,23^. Efavirenz (5, 10 or 20 mg/kg/day), THC (0.75 mg/kg) and MA (methamphetamine hydrochloride (1 mg/kg)) administered on days of drug exposure. The MA and THC dosages were based on previous studies indicating CPP^19,20^. In the sub-acute study animals were exposed to drug (MA, THC or efavirenz) on the mornings of day 1, 3 and 5 and vehicle on days 2, 4 and 6 of drug exposure. Two control groups of animals only received vehicle (saline or olive oil respectively) throughout the 6 days of exposure. These specific vehicles do not induce a place preference in the CPP test^19,24^.

The sub-chronic exposure procedure also alternated between an illicit drug and efavirenz on one day followed by a vehicle exposure on the next day, lasting for 14 days. Thus, the sub-chronic groups received efavirenz, THC or THC + efavirenz on days 1, 3, 5, 7, 9, 11 and 13 and vehicle on days 2, 4, 6, 8, 10, 12, 14, following previous protocols^6,25^. The two vehicle control groups received the respective vehicle exposure throughout the 14 days of exposure.

### Body weight

The body weight of all animals was determined on post-natal day (PND) 21 and again on each day of drug exposure.

### Behavioural analysis

#### Conditioned place preference test (CPP)

The rewarding or aversive properties of a drug can be assessed in the CPP test (reviewed in^25^). The CPP apparatus and paradigm used for this study was adapted from^26^ and validated in our laboratory. Briefly, conditioned testing was conducted in a three-compartment apparatus, constructed of plexiglass and separated by guillotine doors. The two large end compartments (24 × 35 cm) was separated by a smaller centre “choice” compartment (15.5 × 19.5 cm), and used on the habituation and test days. The two outer compartments were visually different and had different floor textures. The CPP paradigm consisted of 3 different stages: (1) a habituation stage (day 1), performed the day before drug exposure, where animals were allowed free access to all 3 compartments for 20 minutes and the compartment in which the least time is spent is then assigned as “the drug-paired compartment” with the most preferred compartment assigned as “the vehicle-paired compartment”^25^; (2) a conditioning stage (days 1–6 for sub-acute or 9–14 for sub-chronic) where animals were injected with either the test drug and subjected to the drug-paired compartment, or vehicle and subjected to the vehicle-paired compartment for 20 minutes; (3) a post-conditioning stage (the day after the last exposure) where animals were allowed free access to all the compartments for 20 minutes, whereupon the time spent in each compartment was assessed. More time spent in the drug-paired compartment (preference) is evidence of a rewarding drug, while less time spent in the drug-paired compartment evidence of an aversive drug^20^.

Behaviour was recorded under dim white light (40 Lux) with a digital video camera and blindly scored using EthoVision© XT software (Noldus Information Technology, Wageningen, Netherlands). The time spent in each compartment prior and post-conditioning was scored. The CPP data is presented as the difference in time spent in the drug-paired compartment utilising the following formula: *Time spent in drug-paired compartment during post-test (s) - Time spent in the drug-paired compartment during habituation (s)*^27^.

#### Open field test (OFT)

The psychomotor stimulant theory of drugs of abuse suggests that most drugs of abuse alter the locomotion of animals (reviewed in^28^). The OFT was performed on day 13 of drug exposure in the sub-chronic study, as described previously^29^. An open field arena (1 m^2^) was illuminated with dim white light (40 Lux) and monitored with a digital video camera. Locomotor activity of the animals (m) was subsequently blindly scored for 10 min using the EthoVision© 225 XT software (Noldus Information Technology, Wageningen, 226 Netherlands). The arena was cleaned with 10% ethanol solution after each test.

#### Sucrose preference test (SPT)

The SPT may indicate whether certain drugs of abuse interact with reward pathways in the brain to promote hedonic activity, i.e. an indulgence in pleasurable activities^30^. The SPT was performed on days 4 and 14 of drug exposure to assess anhedonic or hedonic manifestations in the sub-chronic study, evident in changes in sucrose consumption as previously described^31^. During this test, rats were presented with a free choice between two bottles for 24 h, one containing 0.8% sucrose solution and the other tap water. To eliminate the effects of side preference when drinking, the bottles were changed after 12 hours. Both bottles were weighed to measure the amount of water and sucrose solution consumed after the 24 h period. The preference for sucrose was calculated from the amount of sucrose solution consumed, expressed as a percentage of the total amount of liquid consumed (adapted from^31^).

### Neurochemical and peripheral analyses

#### Blood collection

Trunk blood was collected in pre-chilled heparin tubes, centrifuged at 20 000 x g at 4 °C for 10 min and the plasma stored at −80 °C until the day of analysis^32^.

*Superoxide dismutase (SOD):* Analysis of %SOD in plasma was performed using an SOD activity assay kit (BioVision^TM^ Superoxide Dismutase activity assay kit, California, USA, catalogue number: K335-100), utilising water-soluble tetrazolium (WST)-1 to form a dye following reduction with the superoxide anion. The rate of reduction by sample superoxide is directly related to xanthine oxidase (XO) activity which is inhibited by SOD. The absorbance was read at 450 nm using a Bio-Tek FL600 Microplate Fluorescence Reader (Bio-Tek, Instruments, Inc., 381 Highland Park, Winooski, VT, USA).

#### Brain dissection

Fresh brain tissue was used for the macro-dissection of the frontal cortex, striatum and hippocampus on an ice-cold slab. These brain regions were first identified according to stereological coordinates and subsequently fixed in relation to anatomical landmarks as previously described^33^. The frontal cortex, striatum and hippocampus were snap frozen in liquid nitrogen and stored at −80 °C until the day of monoamines and lipid peroxidation analysis.

Lipid peroxidation: Regional brain lipid peroxidation was assayed using a thiobarbituric acid reactive substances (TBARS) assay kit (Parameter™ thiobarbituric acid reactive substances (TBARS) assay from R&D Systems (Minneapolis, USA; catalogue number KGE013)). Malondialdehyde (MDA) was measured as TBARS, with the total amount of MDA expressed as MDA (µM) and read at 532 nm using a spectrophotometric microplate reader.

Regional brain monoamines: Quantification of frontal cortical, striatal and hippocampal DA, 5-HT and respective metabolites (3,4-dihydroxyphenylacetic acid (DOPAC) and 5-hydroxyindoleacetic acid (5-HIAA)) as well as NA was performed using a high-performance liquid chromatography (HPLC) system with electrochemical detection (HPLC-EC), previously validated in our laboratory^34^. The metabolite of NA, 3-methoxy-4-hydroxyphenylglycol (MHPG) was below the limit of detection and therefore not quantified. Sample monoamine concentrations were determined by comparing the area under the peak to that of the internal standard (isoprenaline). All monoamine concentrations were expressed as ng/mg wet weight of brain tissue^18^. The DA and 5-HT turnover ratios were calculated as DOPAC/DA and 5-HIAA/5-HT, respectively^35^.

### Statistical analysis

Graphpad Prism version 7 for windows (Graphpad software, San Diego, USA) and SAS/STAT® Software were used for the statistical analysis and graphical presentations. Histograms, Q-Q plots and the Shapiro Wilk test were used to test for normality of all the data sets. Animal body weight (mean ± SEM) was analysed by two-way analysis of variance (ANOVA) (with drug exposure and days of weight as the two factors) with repeated measures for different days of weight measured followed by Bonferroni post-hoc analyses. To compare three or more exposure groups, one-way ANOVA was used followed by appropriate post-hoc testing using Dunnett’s or Bonferroni multiple comparison. The nonparametric Kruskal-Wallis test was used if the assumptions of ANOVA were violated. Statistical analysis of the sucrose preference test was performed by two-way ANOVA (with drug exposure and days of testing as the two factors) with repeated measures for different sucrose preference days (days 4 and 14) followed by Bonferroni post-hoc analyses. In all cases data are expressed as the mean ± standard error of the mean (SEM), with a p value of <0.05 deemed statistically significant.

## Results

A comparative analysis done for all the behavioural, neurochemical and peripheral markers on the two vehicle groups returned no significant differences (data not shown), whereupon the saline vehicle and olive oil vehicle groups were pooled and hereafter referred to jointly as the “vehicle” group.

### Body weight

All the exposure groups indicated significant and equal growth over the sub-acute and sub-chronic study periods with no significant group differences observed with respect to drug (efavirenz, MA, THC and THC + efavirenz) or vehicle (saline and olive oil) exposure (data not shown).

### Behavioural analysis

#### Conditioned place preference (CPP)

**Figure 2:**
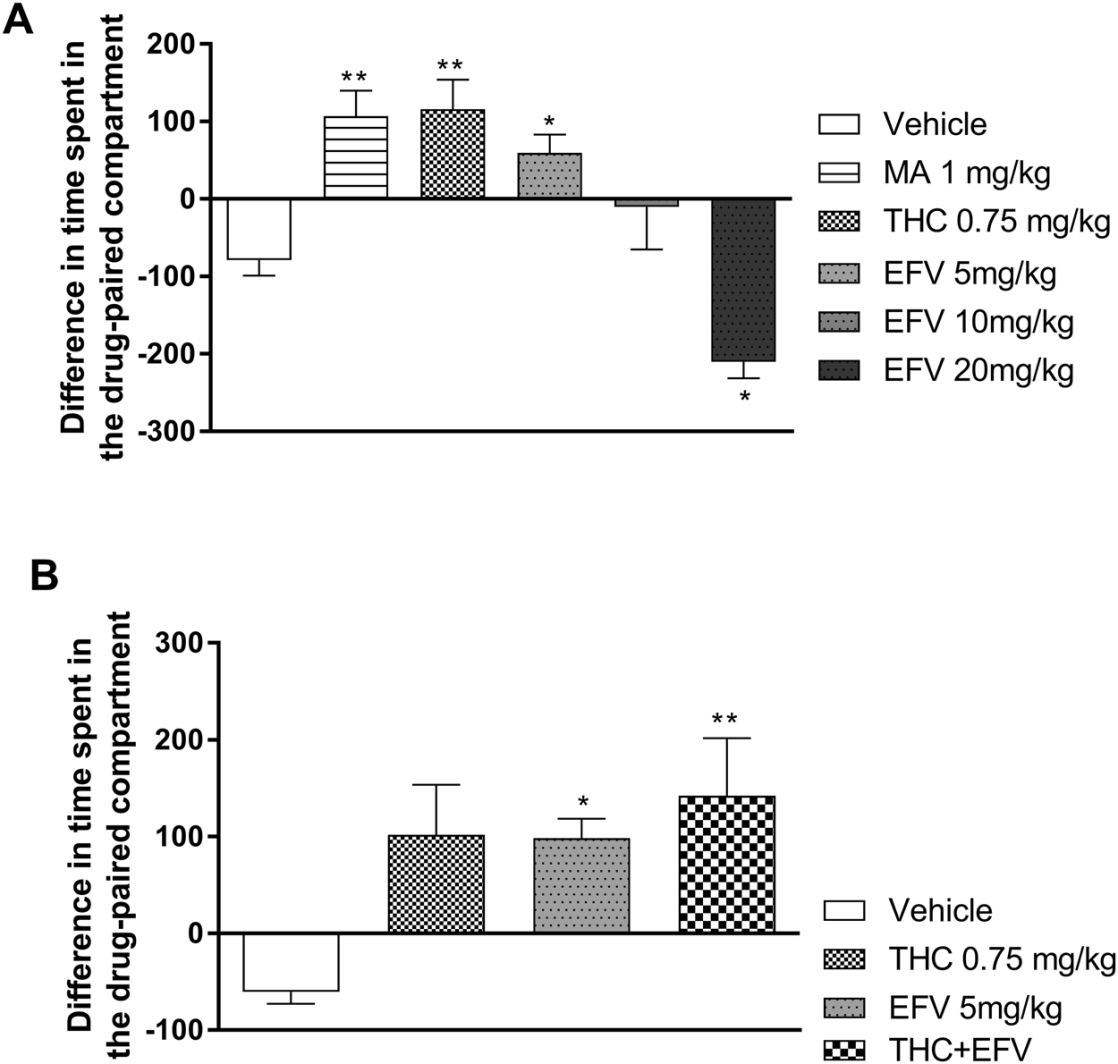
Conditioned place preference (CPP) test in (**A**) the sub-acute and (**B**) the sub-chronic exposure groups. Data presented as difference in time spent in drug-paired compartment: Time spent during the habituation in the drug-paired compartment (s) – time spent during the post-test in the drug-paired compartment (s). Tetrahydrocannabinol (THC); Efavirenz (EFV); Methamphetamine (MA). *p < 0.05, **p < 0.01, vs. Vehicle (One-way ANOVA, Dunnett’s multiple comparison (sub-acute study) and Bonferroni’s post hoc test (sub-chronic study)).

Sub-acute study: One-way ANOVA revealed a significant main effect of drug exposure (F (5, 66) = 1.39, p < 0.0001). Dunnett’s post-hoc multiple comparison revealed that animals exposed to sub-acute MA (1 mg/kg), THC (0.75 mg/kg) and efavirenz at 5 mg/kg presented with a significant increase in the difference in time spent in the drug-paired compartment compared to the vehicle control group (p = 0.0013, 0.0007 and 0.02, respectively) (Fig. 2A). Animals exposed to sub-acute 10 mg/kg efavirenz displayed no place preference compared to the vehicle control group (p = 0.47) whilst animals exposed to sub-acute 20 mg/kg efavirenz showed a significant decrease in place preference compared to the vehicle control group (p = 0.035) (Fig. 2A).

Sub-chronic study: One-way ANOVA revealed a significant main effect of drug exposure (F (3, 36) = 5.52, p = 0.01). Bonferroni post-hoc multiple comparison indicated a significant increase in place preference in animals sub-chronically exposed to 5 mg/kg efavirenz (p = 0.03) and THC + efavirenz (p = 0.005) compared to the vehicle control group (Fig. 2B), while the latter was no different vs. each drug separately. THC alone (0.75 mg/kg) tended to do the same but did not significantly affect place preference in the CPP test (Fig. 2B).

#### Open field test (OFT) (sub-chronic study)

No significant effect of drug exposure between any of the drug exposure groups on locomotor activity were observed (data not shown).

#### Sucrose preference test (SPT) (sub-chronic study)

**Figure 3:**
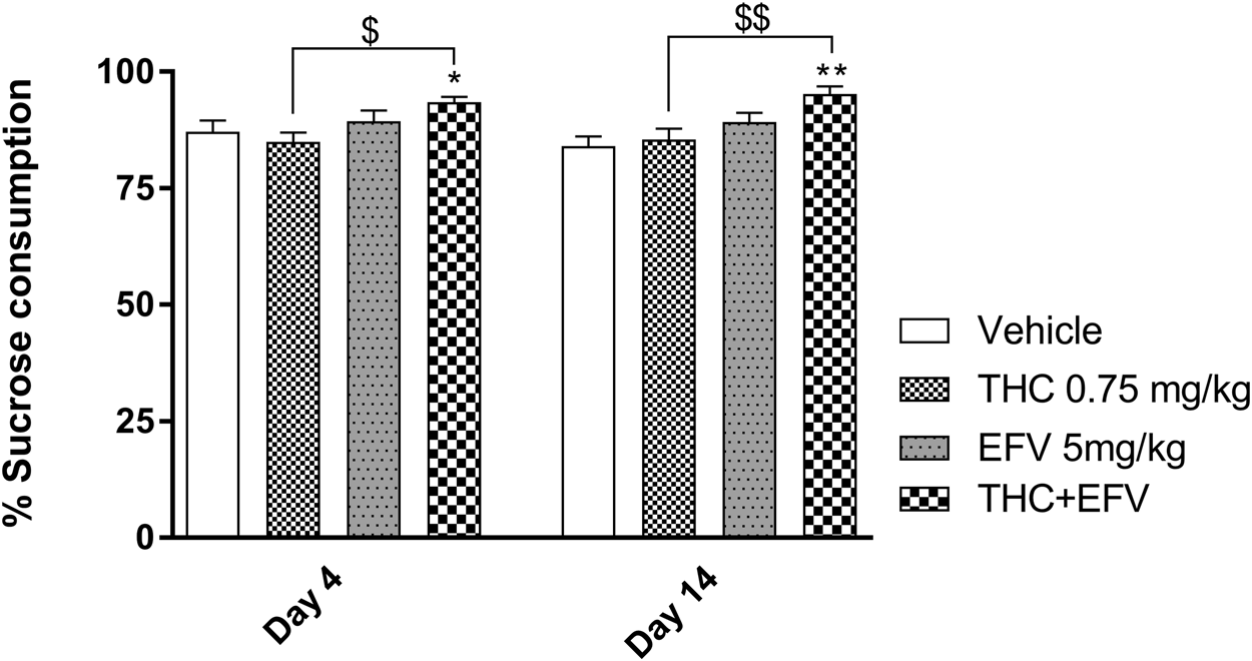
% Sucrose consumption in the sub-chronic drug exposure groups on day 4 and 14 of drug exposure. Tetrahydrocannabinol (THC); Efavirenz (EFV); Methamphetamine (MA). *p < 0.05, **p < 0.01 vs. Vehicle; ^$^p < 0.05, ^$$^p < 0.01 vs. THC (Two-way ANOVA with repeated measures, Bonferroni’s post-hoc test).

A two-way ANOVA with repeated measures indicated a significant main effect of drug exposure (F (3, 44) = 8.63, p = 0.0001) but no significant effect of days (day 4 and 14) (F (1, 44) = 0.03, p = 0.86) on sucrose preference. Bonferroni post hoc test indicated a significant increase in the percentage sucrose consumed between the sub-chronic THC + efavirenz exposed group vs. the vehicle control group on day 4 (p = 0.03) and day 14 (p = 0.001) as well as versus the THC group on day 4 (p = 0.02) and day 14 (p = 0.006) (Fig. 3).

### Peripheral analysis (sub-chronic study)

#### Percentage plasma superoxide dismutase activity (%SOD)

**Figure 4:**
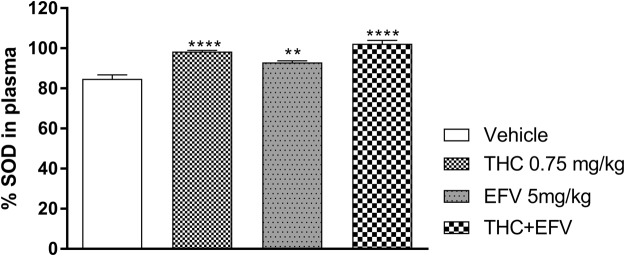
Peripheral percentage superoxide dismutase (%SOD) activity in the sub-chronic drug exposure groups. Tetrahydrocannabinol (THC); Efavirenz (EFV); Methamphetamine (MA). **p < 0.01, ****p < 0.0001, vs Vehicle (One-way ANOVA, Bonferroni’s post-hoc test).

One-way ANOVA revealed a significant main effect of drug exposure on the %SOD (F (3, 44) = 29.21, p < 0.0001). Bonferroni post-hoc analysis indicated a significant increase in %SOD in the sub-chronic THC (p < 0.0001), efavirenz (p = 0.0009) and THC + efavirenz (p < 0.0001) exposed groups compared to the vehicle control (Fig. 4), although no difference was noted in combination exposed animals vs. each drug separately.

### Neurochemical analysis (sub-chronic study)

#### Regional brain lipid peroxidation

One-way ANOVA revealed that drug exposure had a significant main effect on frontal cortical (F (3, 44) = 5.99, p = 0.001) and striatal (F (3, 44) = 8.19, p = 0.0002) but not hippocampal (F (3, 44) = 1.1, p = 0.35) lipid peroxidation (measured as MDA levels).

**Figure 5:**
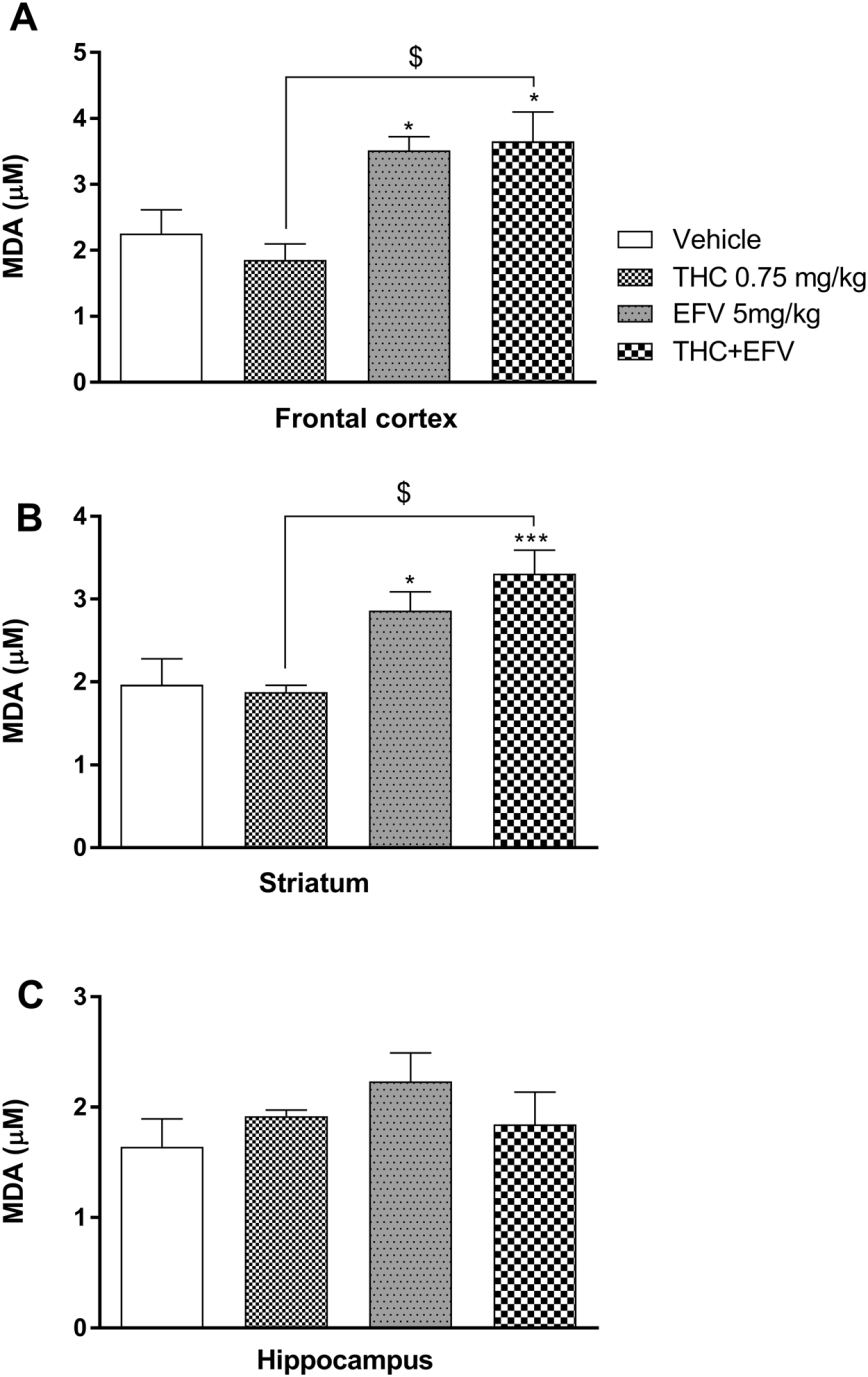
Lipid peroxidation as malondialdehyde (MDA) levels in (**A**) the frontal cortex, (**B**) the striatum and (**C**) the hippocampus in the sub-chronic drug exposure groups. Tetrahydrocannabinol (THC); Efavirenz (EFV); Methamphetamine (MA). *p < 0.05, ***p < 0.001, vs Vehicle, ^$^P < 0.05 vs THC (One-way ANOVA, Bonferroni’s post-hoc test).

Bonferroni post hoc testing indicated that frontal cortical and striatal lipid peroxidation was significantly elevated in animals exposed to sub-chronic efavirenz compared to the vehicle control group (p = 0.047 and p = 0.041, respectively) (Fig. 5A, B). Bonferroni post-hoc analysis also indicated that animals exposed to sub-chronic THC + efavirenz had a significant elevation in lipid peroxidation in the frontal cortex (p = 0.03) and striatum (p = 0.002) compared to the vehicle control group and the THC group (p = 0.04 and p = 0.03 respectively) (Fig. 5A, B), although no difference were noted in combination exposed animals vs. efavirenz separately. No significant differences were observed between the sub-chronic drug exposed groups with regards to hippocampal lipid peroxidation (Fig. 5C).

#### Regional brain monoamines

All regional brain monoamine levels in the different exposure groups are shown in Table 1.

**Table 1:** Selected monoamine levels (ng/mg tissue) in regional brain tissue of rats exposed to sub-chronic vehicle (n = 24), efavirenz (5 mg/kg) (n = 12), THC (0.75 mg/kg) (n = 12) and THC + efavirenz respectively.

	Vehicle	Efavirenz (5 mg/kg)	THC (0.75 mg/kg)	THC + efavirenz
*Frontal cortex*				
DA	60.7 ± 27.54	301.2 ± 72.57*	202.9 ± 50.47^$^	452 ± 131.8**
DOPAC	40.63 ± 8.733	149.4 ± 50.51*^$^	76.19 ± 24.42^$^	197.2 ± 35.71****
DOPAC/DA	1.661 ± 0.3234	0.660 ± 0.1998	0.5048 ± 0.26	5.98 ± 5.44
5-HT	191.6 ± 5.873	235.8 ± 9.696*	129.6 ± 9.56**	197.1 ± 20.07
5-HIAA	201.1 ± 11.64	121.2 ± 4.637***	87.45 ± 4.53****	92.35 ± 3.47****
5-HIAA/5-HT	1.067 ± 0.080	0.607 ± 0.666****	0.71 ± 0.06***	0.53 ± 0.057****
NA	227.5 ± 6.304	220 ± 14.83	148.2 ± 14.19***	144.8 ± 12.42***
*Striatum*				
DA	2228 ± 238.7	3107 ± 158.4*	2030 ± 249.3^$^	3445 ± 306.7**
DOPAC	1109 ± 162.4	731 ± 61.48*	350 ± 37.2***	480 ± 69.65***
DOPAC/DA	0.626 ± 0.174	0.249 ± 0.029*	0.2 ± 0.03*	0.15 ± 0.023**
5-HT	256.8 ± 18.7	366.8 ± 28.02***	162.1 ± 11.39^$$^	339.1 ± 33.29**
5-HIAA	243.5 ± 13.42	260 ± 8.978	151.9 ± 17.16***	144 ± 9.97***
5-HIAA/5-HT	1.027 ± 0.131	0.7991 ± 0.1325	0.97 ± 0.12	0.5 ± 0.07**
NA	81.95 ± 6.985	137.6 ± 15.99*	86.88 ± 9.55	132.6 ± 17.32*
*Hippocampus*				
DA	794.8 ± 223.7	189.5 ± 63.72**	309.1 ± 74.08*	47.24 ± 11.36***
DOPAC	384.5 ± 69.62	152 ± 31.84**	42.77 ± 18.77****	105.3 ± 32.41***
DOPAC/DA	0.823 ± 0.197	1.93 ± 0.490	0.18 ± 0.07^$^	3.19 ± 1.42*
5-HT	234.2 ± 30.91	264.6 ± 18.71	110.6 ± 18.95**	111.9 ± 18.67**
5-HIAA	250.2 ± 13.26	205.2 ± 7.501	154.8 ± 13.38***	123 ± 7.76****
5-HIAA/5-HT	1.323 ± 0.227	0.803 ± 0.042	2.07 ± 0.48	1.7 ± 0.44
NA	279.9 ± 26.74	345.9 ± 55.9	173.3 ± 17.78	321.6 ± 70.37

Presented as Mean ± standard error of the mean (SEM). DA, Dopamine; DOPAC, 3,4-Dihydroxyphenylacetic acid (DOPAC); 5-HT, Serotonin; 5-Hydroxyindoleacetic acid (5-HIAA), NA, Noradrenaline. *p < 0.05, **p < 0.01 vs Vehicle; ^$^p < 0.05, ^$$^p < 0.01 vs THC + efavirenz (One-way ANOVA, Bonferronni post-hoc analysis).

One-way ANOVA revealed a significant main effect of drug exposure on: Frontal cortical DA (F (3,44) = 4.39, p = 0.008), DOPAC (F (3, 44) = 8.03, p = 0.0002), 5-HT (F (3, 44) = 12.4, p < 0.0001), 5-HIAA (F (3, 44) = 59.18, p < 0.0001), 5-HIAA/5HT (F (3,44) = 3.24, p < 0.0001 and NA (F (3, 44) = 13, p < 0.0001); Striatal DA (F (3,44) = 7.7, p = 0.0003), DOPAC (F (3, 44) = 12.28, p < 0.0001), DOPAC/DA (F (3, 44) = 5.66, p = 0.002), 5-HT (F (3, 44) = 14.83, p < 0.0001), 5-HIAA (F (3, 44) = 22.27, p < 0.0001), 5-HIAA/5-HT (F (3, 44) = 4.57, p = 0.007) and NA (F (3, 44) = 4.96, p = 0.005); Hippocampal DA (F (3,44) = 7.42, p = 0.0004), DOPAC (F (3, 44) = 12.24, p < 0.0001), DOPA/DA (F (3, 44) = 3.5, p = 0.03), 5-HT (F (3, 44) = 12.92, p < 0.0001), 5-HIAA (F (3, 44) = 26.63, p < 0.0001) and NA (F (3, 44) = 2.56, p = 0.067).

Frontal cortex (Table 1, top panel): Bonferroni multiple comparisons indicated that: sub-chronic efavirenz significantly increased DA (p = 0.048), DOPAC (p = 0.01) and 5-HT (p = 0.049) and significantly decreased 5-HIAA (p < 0.0001) and 5-HIAA/5-HT (p < 0.0001). Sub-chronic THC significantly decreased 5-HT (p = 0.006), 5-HIAA (p < 0.0001), 5-HIAA/5-HT (p = 0.0005) and NA (p = 0.0003). Sub-chronic THC + efavirenz significantly increased DA (p = 0.007) and DOPAC (p = 0.0002) and significantly decreased 5-HIAA (p < 0.0001), 5-HIAA/5-HT (p < 0.0001) and NA (p = 0.0001), this all in comparison to the vehicle control group. Compared to the sub-chronic THC + efavirenz group, Bonferroni multiple comparison indicated significantly lower frontal cortical DOPAC in the sub-chronic efavirenz group (p = 0.047) and significantly lower frontal cortical DA (p = 0.04) and DOPAC (p = 0.005) in the THC group.

Striatum (Table 1, middle panel): Bonferroni multiple comparisons indicated that: sub-chronic efavirenz significantly increased DA (p = 0.048), 5-HT (p = 0.002) and NA (p = 0.03) and significantly decreased DOPAC (p = 0.044) and DOPAC/DA (p = 0.03). Sub-chronic THC significantly decreased DOPAC (p < 0.0001), DOPAC/DA (p = 0.01), and 5-HIAA (p < 0.0001). Sub-chronic THC + efavirenz significantly increased DA (p = 0.006) and 5-HT (p = 0.02) and NA (p = 0.047) and significantly decreased DOPAC (p = 0.0002), DOPAC/DA (p = 0.004) 5-HIAA (p < 0.0001) and 5-HIAA/5-HT (p = 0.004), this all in comparison to the vehicle control group (Table 1, middle panel). Bonferroni multiple comparison indicated significantly lower striatal DA (p = 0.001) and 5-HT (p < 0.0001) in the sub-chronic THC group compared to the sub-chronic THC + efavirenz group.

Hippocampus (Table 1, bottom panel): Bonferroni multiple comparisons indicated that: sub-chronic efavirenz significantly decreased DA (p = 0.005) and DOPAC (p = 0.002). Sub-chronic THC significantly decreased DA (p = 0.04), DOPAC (p < 0.0001), 5-HT (p = 0.002) and 5-HIAA (p < 0.0001). Sub-chronic THC + efavirenz significantly decreased DA (p = 0.0003), DOPAC (p = 0.0002), 5-HT (p = 0.002) and 5-HIAA (p < 0.0001) and increased DOPAC/DA (p = 0.04), all in comparison to the vehicle control group (Table 1, bottom panel). Compared to the sub-chronic THC + efavirenz group, Bonferroni multiple comparison indicated significantly lower hippocampal DOPAC/DA in the sub-chronic THC group (p = 0.045). No significant differences in hippocampal NA levels were observed between the various exposure groups.

## Discussion

This study demonstrates that sub-acute efavirenz (5 mg/kg) significantly increases CPP in rats, similar to that induced by sub-acute MA (1 mg/kg) and THC (0.75 mg/kg). Moreover, higher doses of efavirenz either fail to induce place preference or are frankly aversive. Efavirenz (5 mg/kg) also induced place preference after sub-chronic exposure, together with increased cortico-striatal DA and 5-HT, increased striatal NA, and elevated oxidative stress markers in plasma and brain. THC and  efavirenz evoked similar behavioural and redox responses as well as increased cortico-striatal DA, 5-HT and NA levels, and a reduction in the hippocampus. Only combined sub-chronic THC + efavirenz exposure was hedonic in the SPT, while sub-chronic THC + efavirenz also induced greater cortico-striatal lipid peroxidation, sucrose preference and increase frontal cortical DA and striatal DA and 5-HT vs. THC alone. Neither efavirenz, THC nor efavirenz + THC markedly affected locomotor behaviour.

Considering the sub-acute study data, efavirenz produced place preference only at lower doses (5 mg/kg), as did THC. No other acute or sub-acute studies on efavirenz at this dosage are available for comparison. However, THC has been found to produce a place preference at very low dosages (0.075–0.75 mg/kg)^36,37^, while higher dosages (eg. 6 mg/kg) are aversive^37^, again not unlike that observed here with the 20 mg/kg dose of efavirenz. Similarly, lower dosages of lysergic acid diethylamide (LSD) (0.2 mg/kg) are more rewarding than higher doses (>0.2 mg/kg)^6,38,39^. The described aversive responses are probably linked to untoward side effects evoked by higher dosages of these drugs^36–38,40,41^.

Importantly, sub-chronic (14 days exposure) efavirenz (5 mg/kg) not only induced sustained rewarding effects, but concomitant disturbances in cortico-striatal monoamines as well as profound redox disturbances in blood and brain was also evident (see below). This finding signifies long-lasting neuro-adaptive and neurotoxic changes that very likely underlie the aforementioned drug-seeking behaviour. Indeed, long-lasting bio-behavioural changes are typical of prolonged exposure to drugs of abuse^42–44^. Interestingly, Gatch *et al*.^6^ did not demonstrate a CPP after sub-acute efavirenz exposure, although differences in dose and duration of exposure may explain such discrepancies, viz. 5 mg/kg or escalating dosages of 10–20 mg/kg for the CPP conditioning sessions. As noted earlier, the rewarding effects of efavirenz at 5 mg/kg may have been rewarding but was lost at higher doses. Previous studies have indicated that the rewarding effects of THC in the CPP test are dose and time (duration of exposure) dependent^37^, possibly explaining the failure of sub-chronic THC to produce a place preference here, although a tendency towards drug-seeking remains evident. Nevertheless, sub-chronic exposure to combined THC and efavirenz induced a significant place preference, although superiority over either drug alone was not observed.

Drug-seeking behaviour, which involves actively *seeking* the drug, is often observed as a secondary manifestation with drugs of abuse (reviewed in^28^). Interestingly, we observed no locomotor changes in efavirenz (5 mg/kg), THC and THC + efavirenz exposed animals in the sub-chronic study. In fact, a recent study^16^ also came to the same conclusion concerning acute or sub-chronic efavirenz, despite exploring higher dosages (25 and 50 mg/kg). Moreover, locomotor activity following cannabinoid administration is either unaltered^45^ or suppressed at high (20–30 mg/kg) but not low dosages (0.75 mg/kg)^46^ of THC^47^, thus emphasizing the variable nature of this parameter. Thus, despite the psychomotor stimulant theory of drugs of abuse^28^, sub-chronic efavirenz and THC + efavirenz does not directly impact locomotor activity as a secondary manifestation. Nevertheless, the basis for a lack of effect on locomotor activity requires further study.

Although often used in assessing anhedonia as a co-presenting symptom of depression (e.g.^30^), the SPT is not an addiction or reward test *per se* but predicts an involvement of the brain DA reward pathways^30^. Despite inducing anhedonia and depression in clinical populations^48–50^, sub-chronic efavirenz (5 mg/kg), failed to alter sucrose preference. Similarly, for THC (0.75 mg/kg), thus in agreement with other studies^51^. However, THC + efavirenz significantly increased sucrose consumption at both days 4 and 14 vs. vehicle and THC alone, suggesting that hedonic effects may be bolstered when the two drugs are combined. This finding is particularly relevant, given the presence of cannabis in efavirenz mixtures (eg. Nyaope and Woonga)^9^ and may explain its popularity among abusers.

Hedonia/anhedonia are closely linked to increased/decreased activity of the DA reward pathways^30,31^, with reduced DA in the frontal cortex linked to a decrease in experiencing pleasure^30,52^. Drugs of abuse specifically activate hedonic “hotspots” in the rat nucleus accumbens, ventral pallidum and prefrontal cortex, with increased DA release necessary for the incentive motivation of hedonia^53^. The current study would therefore support this observation, with increased frontal cortical DA and DOPAC noted in rats subjected to sub-chronic efavirenz (5 mg/kg) and even more so after sub-chronic THC + efavirenz exposure, the latter also presenting with increased sucrose consumption, again hinting at a bolstering action in combined exposure. Incidentally, increased sucrose consumption is also evident with cocaine^54^ and amphetamines, which are known mobilizers of DA in the above brain regions^55^. THC + efavirenz noted here thus pairs with a natural reward (sucrose) to activate brain reward mechanisms that cause cross-sensitization^54^. Thus, efavirenz at least establishes hedonic behaviour when combined with THC, with the involvement of frontal cortical DA reward pathways. Although we did not investigate higher aversive doses of efavirenz (e.g. 20 mg/kg), such high doses *increase* depressive-like behaviour in rats^16^, thus supportive of a dose-dependent balance between its rewarding effects (risk of abuse) vs. neuropsychiatric side-effects such as depression and anhedonia^2^.

Redox dysregulation is well-described in patients treated with efavirenz^56,57^. Altered redox in turn alters neurotransmitter release^58^ and is implicated in mood and psychotic disorders^59,60^. Efavirenz treatment is also associated with elevated striatal glutamate levels^16^ which may induce oxidative stress. Alternatively, high dosages of efavirenz are associated with elevated plasma levels of 8-hydroxy-efavirenz, a known pro-oxidant thought to underlie the neuropsychiatric effects of efavirenz^2,56,61^. Moreover, neurotoxicity underlies some of the neuropsychological effects of drugs of abuse^62^, involving for eg. DA mediated formation of O_2_^−^, H_2_O_2_, OH and quinone adducts. This is especially the case in DA rich regions of the brain^15,63^ and where SOD is compromised in its ability to scavenge these reactive intermediates^15^. Indeed, we show here that animals exposed to sub-chronic efavirenz, THC and THC + efavirenz all display significantly increased plasma %SOD activity, although no apparent reinforcing effect is evident in combined THC + efavirenz exposed animals. Moreover, significantly elevated lipid peroxidation was evident in the striatum and frontal cortex, but not the hippocampus, of sub-chronic efavirenz and THC + efavirenz exposed animals, while sub-chronic THC + efavirenz was significantly superior to THC alone regarding regional brain lipid peroxidation, possibly due to THC not increasing cortico-striatal lipid peroxidation alone. Thus, efavirenz exposure increased ROS, this in an attempt to detoxify formed reactive intermediates. Supportive of this, similar findings are noted with cocaine^64^, while efavirenz has also been found to increase endoplasmic reticulum stress and autophagy in human endothelial cells^65^. Importantly, the aforementioned increase in lipid peroxidation co-presented with elevated DA levels in the brain reward areas, viz. frontal cortex and striatum, thus supportive of DA-mediated oxidative stress. However, sub-chronic THC neither increased cortico-striatal DA nor lipid peroxidation levels, observations that correspond with previous preclinical studies^66,67^ and that could explain the lack of augmentation in THC + efavirenz groups vs. efavirenz alone.

DA plays a crucial role in mediating reward and learning^68,69^. Sub-chronic efavirenz at 5 mg/kg and THC + efavirenz significantly increased DA levels in the frontal cortex, involved in reward and learning^70^, and the striatum involved in decision making and reward learning^71^. Moreover, increased frontal cortical DOPAC levels was evident in the THC + efavirenz group, possibly supporting elevated DA in this region. Cortico-striatal D_1_ receptors^72^ drives reward effects as well as the reinforcement of drug seeking behaviour, place preference for drugs of abuse, and enhancement of palatability of food^52,73,74^. Sub-chronic THC, on the other hand, did not affect cortico-striatal DA or place preference. Interestingly, sub-chronic efavirenz, THC and THC + efavirenz significantly decreased hippocampal DA levels (implicated in memory and reward anticipation), a region rich in high affinity D_2_ receptors^75^ responsible for maintaining drug-seeking motivation^11,52^. However increased hippocampal DA turnover in sub-chronic THC + efavirenz groups indicates an increase in DA metabolism, possibly explaining lower DA levels in this region and in line with previous efavirenz studies^16,71^. Moreover, reduced DA turnover in the striatum after sub-chronic efavirenz, THC and THC + efavirenz exposure suggests diminished DA metabolism via monoamine oxidase (MAO)^15^ and thus an increase in DA bioavailability, evinced by the higher DA levels in this brain region, although not significantly so in the THC group. Incidentally, Gatch *et al*.^6^ describe interactions between efavirenz and DA transporters, which may also explain the observed dopaminergic effects in efavirenz- and efavirenz + THC exposed animals.

Drugs of abuse such as LSD, cocaine and MA interact with 5HT_2_ receptors or 5-HT transporters, respectively^76^, to increase frontal-cortical DA and 5-HT levels^77^. 5-HT is also involved in emotion and memory processes pertaining to drug addiction^76–78^. Sub-chronic efavirenz increased frontal cortical and striatal 5-HT levels without noticeable effects on hippocampal 5-HT. Paradoxically, sub-chronic THC *reduced* frontal cortical 5-HT, possibly also contributing towards unaltered frontal cortical 5-HT observed in the sub-chronic THC + efavirenz group, a finding that may explain THC induced cognitive and memory deficits in rodents (reviewed in^79^). Recently, Gatch *et al*., 2013 described interactions between efavirenz and 5-HT transporters^6^, supportive of how efavirenz may increase 5-HT levels described here. However, sub-chronic THC and THC + efavirenz significantly *reduced* hippocampal 5-HT and 5-HIAA, thus countering the bolstering effect of efavirenz. This might be related to a down regulation of CB_1_ receptors and a resultant up-regulation of 5-HT_2A_ receptors (see review by^80^). Decreased frontal cortical 5-HT turnover in sub-chronic efavirenz, THC and THC + efavirenz groups, and in the striatum of THC + efavirenz exposed rats, suggest increased 5-HT levels due to decreased metabolism, in line with previous studies^16,81^. However, no augmenting effects on 5-HT were observed in the THC + efavirenz group vs. either drug alone.

No changes in hippocampal NA levels were observed in any of the exposure groups, although sub-chronic THC and THC + efavirenz significantly decreased frontal cortical NA while sub-chronic efavirenz and THC + efavirenz significantly increased striatal NA. These responses are not unlike cortical hypoadrenergia and striatal hyperadrenergia that have been associated with cognitive deficits and psychosis, respectively^59^. Regional brain increases in NA might be a unifying etiological factor in drug abuse, viz. suppressing noradrenergic signalling during chronic use but increasing NA levels in reward circuits^82^. Furthermore, low and high doses of THC decrease and increase frontal cortical NA levels, respectively (reviewed in^82^). However, chronic efavirenz exposure has been noted to reduce striatal DA, 5-HT and NA^16^, although other regions were not assessed. That said, the latter findings were observed at much higher dosages, coinciding with the aversive doses described earlier in the CPP test after sub-acute exposure. The reduction in striatal monoamines^16^ possibly correlates with depressive and anxiety-like behaviour associated with efavirenz^2^. Indeed, the neuropsychiatric side effects of efavirenz are directly related to its plasma concentration^3^, with higher dosages more associated with depression^2^. THC + efavirenz did not have augmenting effects on regional NA vs. either drug alone.

## Conclusion

Sub-acute efavirenz (5 mg/kg) provokes drug-seeking behaviour that closely parallels that of MA and THC, with higher doses ineffective or aversive. Sub-chronic efavirenz (5 mg/kg) alone or in combination with THC is similarly addictive and hedonic, as well as increases cortico-striatal DA, 5-HT, lipid peroxidation and peripheral oxidative stress. Sub-chronic THC + efavirenz was significantly superior to THC alone regarding regional brain lipid peroxidation, frontal cortical DA, DOPAC, striatal DA, 5-HT and hippocampal DOPAC/DA alterations, and hedonic behaviour, but superior to efavirenz regarding increased frontal cortical DOPAC, suggesting synergistic effects.

The neuropsychiatric effects of efavirenz are widely evident in the literature, as is the problem of HIV-associated neuropsychiatric disorder (HAND)^2^. Inflammation and altered redox status modifies monoaminergic transmission to contribute to the manifestation of depression, psychosis and addictive behavior^58,59,83^. With efavirenz known to be brain penetrant and to promote the formation of a pro-oxidant metabolite, 8-OH efavirenz, this paper brings together startling evidence that efavirenz-associated redox disturbances and altered limbic brain monoamines lay the foundation for addictive-like behavior. Although measuring redox parameters may represent a simple biomarker to confirm efavirenz-associated neuropsychiatric effects, redox dysfunction characterizes many neuropsychiatric disorders thus complicating any such interpretation^59^. Nevertheless, our data suggests that *targeting* cellular redox biology pharmacologically may reduce or prevent these adverse effects. While it may not always be possible to restrict the use of efavirenz due to its superior efficacy vs. other anti-HIV drugs^1^, adjunctive treatment with a brain and peripherally active antioxidant, such as N-acetyl cysteine (NAC), may offer prophylactic and therapeutic benefits for efavirenz-associated drug seeking behavior.

This work therefore proposes possible mechanisms for efavirenz associated drug-seeking behaviour while also reiterating the abuse potential of efavirenz-cannabis combinations. The findings hint towards the possible treatment of Nyope addiction with antioxidants, which may aid in the development of new strategies to diminish these effects and to improve ART.

## References

[1] Staszewski S (1999). Efavirenz plus zidovudine and lamivudine, efavirenz plus indinavir, and indinavir plus zidovudine and lamivudine in the treatment of HIV-1 infection in adults. N. Engl. J. Med..

[2] Dalwadi, D., Ozuna, L., Harvey, B., Viljoen, M. & Schetz, J. Adverse neuropsychiatric events and recreational use of efavirenz and other HIV-1 antiretroviral drugs. *Pharmacolog rev*. in press (2018). 10.1124/pr.117.013706 29945900

[3] Marzolini C (2001). Efavirenz plasma levels can predict treatment failure and central nervous system side effects in HIV-1-infected patients. AIDS.

[4] Kryst J, Kawalec P, Pilc A (2015). Efavirenz-based regimens in antiretroviral-naive HIV-infected patients: a systematic review and meta-analysis of randomized controlled trials. PloS one.

[5] Almond LM, Hoggard PG, Edirisinghe D, Khoo SH, Back DJ (2005). Intracellular and plasma pharmacokinetics of efavirenz in HIV-infected individuals. J. Antimicrob. Chemother..

[6] Gatch MB (2013). The HIV antiretroviral drug efavirenz has LSD-like properties. Neuropsychopharmacol.

[7] Schmitt KC, Reith ME (2010). Regulation of the dopamine transporter. Ann. N. Y. Acad. Sci..

[8] Sora I (2001). Molecular mechanisms of cocaine reward: combined dopamine and serotonin transporter knockouts eliminate cocaine place preference. Proc. Natl. Acad. Sci. USA.

[9] Mokwena KE (2015). The Novel Psychoactive Substance ‘Nyaope’Brings Unique Challenges to Mental Health Services in South Africa. Int J Emer Mental Health Human Resilience.

[10] Yagura H (2017). Effect of dolutegravir plasma concentration on central nervous system side effects. Anxiety.

[11] Goldstein RZ, Volkow ND (2002). Drug addiction and its underlying neurobiological basis: neuroimaging evidence for the involvement of the frontal cortex. Am. J. Psychiatry.

[12] Patkar, O. L., Belmer, A. & Bartlett, S. E. Recent Advances in Drug Addiction Research and Clinical Applications. Chapter 5: Contribution of Noradrenaline, Serotonin, and the Basolateral Amygdala to Alcohol Addiction: Implications for Novel Pharmacotherapies for AUDs (2016).

[13] Gibbs ME, Summers RJ (2002). Role of adrenoceptor subtypes in memory consolidation. Prog. Neurobiol..

[14] Müller CP, Carey RJ, Huston JP, Silva MADS (2007). Serotonin and psychostimulant addiction: focus on 5-HT1A-receptors. Prog. Neurobiol..

[15] Cunha-Oliveira T, Rego AC, Oliveira CR (2008). Cellular and molecular mechanisms involved in the neurotoxicity of opioid and psychostimulant drugs. Brain Res. Rev..

[16] Cavalcante GIT (2017). HIV antiretroviral drug Efavirenz induces anxiety-like and depression-like behavior in rats: evaluation of neurotransmitter alterations in the striatum. Eur. J. Pharmacol..

[17] Kilkenny C, Browne W, Cuthill IC, Emerson M, Altman DG (2010). Animal research: reporting *in vivo* experiments: the ARRIVE guidelines. Br. J. Pharmacol..

[18] Möller M (2013). Social isolation rearing induces mitochondrial, immunological, neurochemical and behavioural deficits in rats, and is reversed by clozapine or N-acetyl cysteine. Brain Behav. Immun..

[19] Zakharova E, Leoni G, Kichko I, Izenwasser S (2009). Differential effects of methamphetamine and cocaine on conditioned place preference and locomotor activity in adult and adolescent male rats. Behav. Brain Res..

[20] Braida D, Iosue S, Pegorini S, Sala M (2004). Δ9-Tetrahydrocannabinol-induced conditioned place preference and intracerebroventricular self-administration in rats. Eur. J. Pharmacol..

[21] Iturria SJ (2011). A method for obtaining randomized block designs in preclinical studies with multiple quantitative blocking variables. Pharmaceut stat.

[22] Sharma P, Murthy P, Bharath MM (2012). Chemistry, metabolism, and toxicology of cannabis: clinical implications. Iran. J. Psychiatry..

[23] Gaur PK, Mishra S, Bajpai M, Mishra A (2014). Enhanced oral bioavailability of efavirenz by solid lipid nanoparticles: *in vitro* drug release and pharmacokinetics studies. Biomed. Res. Int..

[24] da Silveira NS, de Oliveira-Silva GL, de Freitas Lamanes B (2014). da Silva Prado, Ligia Carolina & Bispo-da-Silva, L. B. The aversive, anxiolytic-like, and verapamil-sensitive psychostimulant effects of pulegone. Biol Pharmaceut Bull.

[25] Bardo M, Bevins RA (2000). Conditioned place preference: what does it add to our preclinical understanding of drug reward?. Psychopharmacology (Berl.).

[26] Tzschentke TM (2001). Pharmacology and behavioral pharmacology of the mesocortical dopamine system. Prog. Neurobiol..

[27] Malanga C, Pejchal M, Kosofsky BE (2007). Prenatal exposure to cocaine alters the development of conditioned place-preference to cocaine in adult mice. Pharmacol Biochem Behav.

[28] Weinshenker D, Schroeder JP (2007). There and back again: a tale of norepinephrine and drug addiction. Neuropsychopharmacology.

[29] Gould, T. D., Dao, D. T. & Kovacsics, C. E. The open field test. *Mood and anxiety related phenotypes in mice: Characterization using behavioral tests*, 1–20 (2009).

[30] Der-Avakian A, Markou A (2012). The neurobiology of anhedonia and other reward-related deficits. Trends Neurosci..

[31] Rygula R (2005). Anhedonia and motivational deficits in rats: impact of chronic social stress. Behav. Brain Res..

[32] Möller M, Du Preez JL, Emsley R, Harvey BH (2011). Isolation rearing-induced deficits in sensorimotor gating and social interaction in rats are related to cortico-striatal oxidative stress, and reversed by sub-chronic clozapine administration. Eur. Neuropsychopharmacol..

[33] Spijker, S. In *Neuroproteomics* (ed. Li, K. W.) 13–26 (Springer, 2011).

[34] Harvey BH, Brand L, Jeeva Z, Stein DJ (2006). Cortical/hippocampal monoamines, HPA-axis changes and aversive behavior following stress and restress in an animal model of post-traumatic stress disorder. Physiol. Behav..

[35] Gemmel M (2016). Developmental fluoxetine and prenatal stress effects on serotonin, dopamine, and synaptophysin density in the PFC and hippocampus of offspring at weaning. Dev. Psychobiol..

[36] Pistis M (2002). Δ9-Tetrahydrocannabinol decreases extracellular GABA and increases extracellular glutamate and dopamine levels in the rat prefrontal cortex: an *in vivo* microdialysis study. Brain Res..

[37] Cheer J, Kendall D, Marsden C (2000). Cannabinoid receptors and reward in the rat: a conditioned place preference study. Psychopharmacology (Berl.).

[38] Meltzer HY, Fessler RG, Simonovic M, Doherty J, Fang VS (1977). Lysergic acid diethylamide: evidence for stimulation of pituitary dopamine receptors. Psychopharmacology (Berl.).

[39] Passie T, Halpern JH, Stichtenoth DO, Emrich HM, Hintzen A (2008). The pharmacology of lysergic acid diethylamide: a review. CNS Neurosci Therap.

[40] Goodwin AK (2016). An intravenous self-administration procedure for assessing the reinforcing effects of hallucinogens in nonhuman primates. J. Pharmacol. Toxicol. Methods.

[41] Cespedes MS, Aberg JA (2006). Neuropsychiatric complications of antiretroviral therapy. Drug safety.

[42] Do Couto BR, Aguilar M, Rodriguez-Arias M, Minarro J (2005). Long-lasting rewarding effects of morphine induced by drug primings. Brain Res..

[43] Valjent E (2006). Plasticity-associated gene Krox24/Zif268 is required for long-lasting behavioral effects of cocaine. J. Neurosci..

[44] Kenny PJ, Markou A (2006). Nicotine self-administration acutely activates brain reward systems and induces a long-lasting increase in reward sensitivity. Neuropsychopharmacology.

[45] Khani A (2015). Activation of cannabinoid system in anterior cingulate cortex and orbitofrontal cortex modulates cost-benefit decision making. Psychopharmacology (Berl.).

[46] Rubino T (2007). Cellular mechanisms underlying the anxiolytic effect of low doses of peripheral Δ 9-tetrahydrocannabinol in rats. Neuropsychopharmacology.

[47] Taffe MA, Creehan KM, Vandewater SA (2015). Cannabidiol fails to reverse hypothermia or locomotor suppression induced by Δ9‐tetrahydrocannabinol in Sprague‐Dawley rats. Br. J. Pharmacol..

[48] Fumaz CR (2002). Quality of life, emotional status, and adherence of HIV-1-infected patients treated with efavirenz versus protease inhibitor-containing regimens. Jaids-Hagerstown Md.

[49] Fumaz CR (2005). Long-term neuropsychiatric disorders on efavirenz-based approaches: quality of life, psychologic issues, and adherence. *JAIDS J. Acquired Immune Defic*. Syndromes.

[50] Blanch J (2001). Preliminary data of a prospective study on neuropsychiatric side effects after initiation of efavirenz. J. Acquir. Immune Defic. Syndr..

[51] Brown JE, Kassouny M, Cross JK (1977). Kinetic studies of food intake and sucrose solution preference by rats treated with low doses of Δ9-tetrahydrocannabinol. Behav. Biol..

[52] Graham DL, Hoppenot R, Hendryx A, Self DW (2007). Differential ability of D1 and D2 dopamine receptor agonists to induce and modulate expression and reinstatement of cocaine place preference in rats. Psychopharmacology (Berl.).

[53] Berridge KC, Kringelbach ML (2015). Pleasure systems in the brain. Neuron.

[54] Gosnell BA (2005). Sucrose intake enhances behavioral sensitization produced by cocaine. Brain Res..

[55] Avena NM, Hoebel BG (2003). Amphetamine-sensitized rats show sugar-induced hyperactivity (cross-sensitization) and sugar hyperphagia. Pharmacol Biochem Behav.

[56] Tovar-y-Romo LB (2012). Dendritic spine injury induced by the 8-hydroxy metabolite of efavirenz. J. Pharmacol. Exp. Ther..

[57] Harjivan SG (2014). The phenolic metabolites of the anti-HIV drug efavirenz: evidence for distinct reactivities upon oxidation with Frémy’s salt. Eur. J. Med. Chem..

[58] Möller M, Swanepoel T, Harvey BH (2015). Neurodevelopmental Animal Models Reveal the Convergent Role of Neurotransmitter Systems, Inflammation, and Oxidative Stress as Biomarkers of Schizophrenia: Implications for Novel Drug Development. ACS Chem Neurosci.

[59] Brand J, Moller M, Harvey B (2015). A review of biomarkers in mood and psychotic disorders: a dissection of clinical vs. preclinical correlates. Curr Neuropharmacol.

[60] Barron H, Hafizi S, Andreazza AC, Mizrahi R (2017). Neuroinflammation and oxidative stress in psychosis and psychosis risk. Int J Mol Sci.

[61] Decloedt EH, Maartens G (2013). Neuronal toxicity of efavirenz: a systematic review. Expert opinion on drug safety.

[62] Leshner AI (1997). Addiction is a brain disease, and it matters. Sci.

[63] O’Dell SJ, Weihmuller FB, Marshall JF (1991). Multiple methamphetamine injections induce marked increases in extracellular striatal dopamine which correlate with subsequent neurotoxicity. Brain Res..

[64] Dietrich J (2005). Acute or repeated cocaine administration generates reactive oxygen species and induces antioxidant enzyme activity in dopaminergic rat brain structures. Neuropharmacology.

[65] Weiß M (2016). Efavirenz causes oxidative stress, endoplasmic reticulum stress, and autophagy in endothelial cells. Cardiovas Toxicol.

[66] Calderón G, Esquivel G, García E, Osnaya N, Juárez Olguín H (2010). Effects of marijuana and diazepam on lipid peroxidation, Na, K ATPase, and levels of glutathione and 5-HTP in rat brain. Acta. Biol. Hung..

[67] Coskun ZM, Bolkent S (2016). Evaluation of Delta(9)-tetrahydrocannabinol metabolites and oxidative stress in type 2 diabetic rats. Iran. J. Basic Med. Sci..

[68] Goodman A (2008). Neurobiology of addiction: An integrative review. Biochem. Pharmacol..

[69] Koob GF (2000). Neurobiology of addiction: toward the development of new therapies. Ann. N. Y. Acad. Sci..

[70] Kalivas PW, McFarland K (2003). Brain circuitry and the reinstatement of cocaine-seeking behavior. Psychopharmacology (Berl.).

[71] Balleine BW, Delgado MR, Hikosaka O (2007). The role of the dorsal striatum in reward and decision-making. J. Neurosci..

[72] Beaulieu J-M, Gainetdinov RR (2011). The physiology, signaling, and pharmacology of dopamine receptors. Pharmacol. Rev..

[73] Karasinska JM, George SR, Cheng R, O’dowd BF (2005). Deletion of dopamine D1 and D3 receptors differentially affects spontaneous behaviour and cocaine‐induced locomotor activity, reward and CREB phosphorylation. Eur. J. Neurosci..

[74] Cooper S, Al-Naser H (2006). Dopaminergic control of food choice: contrasting effects of SKF 38393 and quinpirole on high-palatability food preference in the rat. Neuropharmacology.

[75] Seeman P (2006). Targeting the dopamine D2 receptor in schizophrenia. Expert opinion on therapeutic targets.

[76] Kirby L, Zeeb F, Winstanley C (2011). Contributions of serotonin in addiction vulnerability. Neuropharmacology.

[77] Müller N, Schwarz M (2007). The immune-mediated alteration of serotonin and glutamate: towards an integrated view of depression. Mol. Psychiatry.

[78] Halberstadt AL, Geyer MA (2013). Characterization of the head-twitch response induced by hallucinogens in mice. Psychopharmacology (Berl.).

[79] Egerton A, Allison C, Brett RR, Pratt JA (2006). Cannabinoids and prefrontal cortical function: insights from preclinical studies. Neurosci Biobehav Rev.

[80] Viñals X (2015). Cognitive impairment induced by delta9-tetrahydrocannabinol occurs through heteromers between cannabinoid CB1 and serotonin 5-HT2A receptors. PLoS biology.

[81] Molina-Holgado F, Molina-Holgado E, Leret ML, González MI, Reader TA (1993). Distribution of indoleamines and [3 H] paroxetine binding in rat brain regions following acute or perinatal Δ 9-tetrahydrocannabinol treatments. Neurochem. Res..

[82] Fitzgerald, P. J. Elevated norepinephrine may be a unifying etiological factor in the abuse of a broad range of substances: alcohol, nicotine, marijuana, heroin, cocaine, and caffeine. *Substance abuse: research and treatment* **7**, SART S13019 (2013). 10.4137/SART.S13019 PMC3798293 24151426

[83] Beardsley PM, Hauser KF (2014). Glial modulators as potential treatments of psychostimulant abuse. Adv Pharmacol..

